# c-Src/Cav1-dependent activation of the EGFR by Dsg2

**DOI:** 10.18632/oncotarget.7675

**Published:** 2016-02-24

**Authors:** Andrew M. Overmiller, Kathleen P. McGuinn, Brett J. Roberts, Felicia Cooper, Donna M. Brennan-Crispi, Takahiro Deguchi, Sirkku Peltonen, Wahl James K., M? G. Mahoney

**Affiliations:** ^1^ Department of Dermatology and Cutaneous Biology, Thomas Jefferson University, Philadelphia, PA, USA; ^2^ Department of Oral Biology, University of Nebraska Medical Center, Lincoln, NE, USA; ^3^ Department of Biochemistry and Molecular Biology, Thomas Jefferson University, Philadelphia, PA, USA; ^4^ Laboratory of Biophysics, Department of Cell Biology and Anatomy, University of Turku, Turku, Finland; ^5^ Department of Dermatology, University of Turku and Turku Hospital, Turku, Finland

**Keywords:** EGFR, c-Src, desmoglein-2, caveolin-1, Stat3

## Abstract

The desmosomal cadherin, desmoglein 2 (Dsg2), is deregulated in a variety of human cancers including those of the skin. When ectopically expressed in the epidermis of transgenic mice, Dsg2 activates multiple mitogenic signaling pathways and increases susceptibility to tumorigenesis. However, the molecular mechanism responsible for Dsg2-mediated cellular signaling is poorly understood. Here we show overexpression as well as co-localization of Dsg2 and EGFR in cutaneous SCCs *in vivo*. Using HaCaT keratinocytes, knockdown of Dsg2 decreases EGFR expression and abrogates the activation of EGFR, c-Src and Stat3, but not Erk1/2 or Akt, in response to EGF ligand stimulation. To determine whether Dsg2 mediates signaling through lipid microdomains, sucrose density fractionation illustrated that Dsg2 is recruited to and displaces Cav1, EGFR and c-Src from light density lipid raft fractions. STED imaging confirmed that the presence of Dsg2 disperses Cav1 from the cell-cell borders. Perturbation of lipid rafts with the cholesterol-chelating agent MβCD also shifts Cav1, c-Src and EGFR out of the rafts and activates signaling pathways. Functionally, overexpression of Dsg2 in human SCC A431 cells enhances EGFR activation and increases cell proliferation and migration through a c-Src and EGFR dependent manner. In summary, our data suggest that Dsg2 stimulates cell growth and migration by positively regulating EGFR level and signaling through a c-Src and Cav1-dependent mechanism using lipid rafts as signal modulatory platforms.

## INTRODUCTION

Desmogleins are transmembrane glycoproteins of the adhesion structures desmosomes. Of particular interest to this study is desmoglein 2 (Dsg2), which is expressed in the basal epidermis, intestinal epithelia, cardiac tissue, and hair follicles [1, 2]. The role of Dsg2 and related desmogleins in desmosome assembly and adhesion is well known, but its role beyond cellular adhesion is an emerging focus of research. In humans, mutations in the Dsg2 gene are the underlying cause of the sudden death condition arrhythmogenic right ventricular cardiomyopathy [3]; Dsg2 serves as a receptor for adenoviruses that are involved in respiratory and urinary tract infections [4]; and Dsg2 has been identified as a regulator of β-amyloid protein processing in Alzheimer's disease [5]. Ectodomain shedding of Dsg2 disrupts intercellular adhesion and promotes cell proliferation to promote wound repair in ulcerative colitis [6]. In mice, Dsg2 gene knockout results in defects in blastocyst proliferation and embryonic lethality [7]. Conversely, expression of Dsg2 in the superficial epidermis of transgenic mice enhances cell proliferation and increases susceptibility to chemical-induced skin carcinogenesis [8].

Dsg2 is markedly increased in skin malignancies including basal and squamous cell carcinoma (BCC and SCC) [1, 9–12]. Altered Dsg2 expression also occurs in prostate and colon cancers, suggesting a role for Dsg2 in oncogenesis in a variety of epithelial tissues [13–16]. Loss of Dsg2 in colonic epithelial carcinoma cells results in decreased proliferation and suppresses xenograft tumor growth in mice [17]. However, in diffuse-type gastric cancers, decreased expression of Dsg2 is associated with poor prognosis suggesting that Dsg2 may have dual roles as an oncogene and a tumor-suppressor gene [18]. The signaling pathways through which Dsg2 exerts its observed oncogenic effects remain to be elucidated. We previously showed that Dsg2 enhances activation of growth and survival pathways, including PI3K/Akt, MEK-Erk1/2, JAK/Stat3 and NF-κB [8] and alters a number of genes important in epithelial dysplasia [19]. Interestingly, these signaling pathways are downstream of the epidermal growth factor receptor (EGFR) and activation with EGF increases association of EGFR binding to Dsg2 [20]. Furthermore, Dsg2 upregulates Hedgehog signaling and in response to chemical carcinogens, enhances BCC and SCC tumor development [21].

The mechanism by which Dsg2 modulates signaling may involve its interaction with caveolin-1 (Cav1), an integral membrane protein of caveolar lipid rafts [22–25]. Cav1, through its cytosolic caveolin scaffolding domain, can interact with and sequester a number of different signaling molecules including EGFR [26]. Both tumor growth and anchorage-independent cell survival are negatively impacted by Cav1 overexpression [27, 28]. EGFR-stimulated phosphorylation of tyrosine 14 of Cav1 has been shown to promote caveolae formation in a c-Src-dependent manner, which in turn promotes EGFR sequestration and inactivation [29, 30]. Active signaling through EGFR requires disassociation from caveolae; given Dsg2′s association with Cav1 and the established interactions between Dsg2 and EGFR, we examined the potential role of Dsg2-mediated modulation of EGFR signaling [14, 17, 22, 31–35].

Herein we report that Dsg2 and EGFR expression is upregulated and colocalizes in human SCCs. Knockdown of Dsg2 reduces EGFR level and activation. Furthermore, Dsg2 mobilizes Cav1, EGFR, and c-Src lipid raft localization, altering cell signaling. Additionally, overexpression of Dsg2 enhances proliferation and migration in cancer cells. Taken together, these results reveal a distinct signal-regulating role for Dsg2 beyond its function in cell-cell adhesion.

## RESULTS

### Dsg2 enhances EGFR level and activation

**Figure 1:**
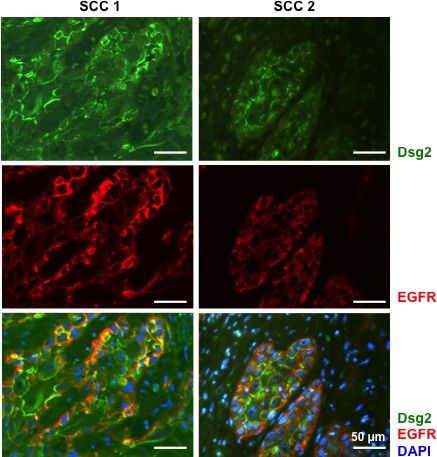
Co-localization of Dsg2 and EGFR in squamous cell carcinomas Two representative SCCs were co-immunostained for Dsg2 (green) and EGFR (red). DAPI to label nuclear DNA (blue). Scale bar = 50 μm.

**Figure 2:**
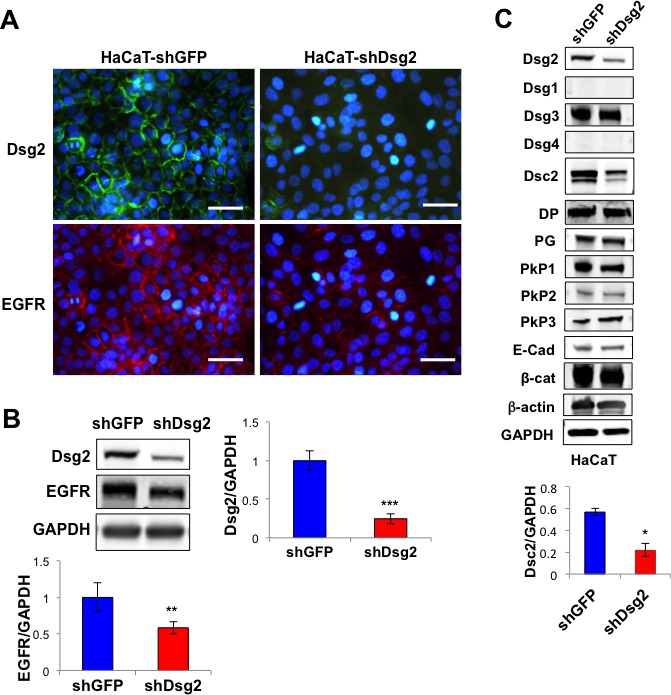
Knockdown of Dsg2 reduces EGFR **A.** HaCaT keratinocytes were stably transfected with shRNA to GFP (shGFP) or Dsg2 (shDsg2) and selected in puromycin. Cells were plated on glass slides and processed for immunofluorescence for Dsg2 (green) and EGFR (red). Blue DAPI counterstain for nuclei. Scale bar = 100 μm. **B.** Total lysates from HaCaT-shGFP and -shDsg2 cells were immunoblotted for Dsg2, EGFR and GAPDH for equal loading. Densitometry was performed and histogram bars represent the relative amount of Dsg2 normalized GAPDH. Data are expressed as average value ± s.e.m. of at least 3 independent experiments. Dsg2 (shGFP, 1.00±0.12; shDsg2, 0.25±0.06); EGFR (shGFP, 1.00±0.20; shDsg2, 0.58±0.09); **p < 0.01; ***p < 0.001; *t*-test. **C.** HaCaT-shGFP and -shDsg2 cells were immunoblotted for Dsg1-4, desmocollin 2 (Dsc2), desmoplakin (DP), plakoglobin (PG), plakophilin 1-3 (PkP1-3), E-cadherin (E-cad), β-catenin (β-cat) and GAPDH. Blotting for β-actin and GAPDP showed equal loading. Densitometry represents the ratio of Dsc2/GAPDH expressed as average value ± standard of the mean. N = 3. Dsc2 (shGFP, 0.57±0.03; shDsg2, 0.22±0.06); *p < 0.05; *t*-test.

Dsg2 and EGFR have been shown to enhance epithelial cell growth and survival and are overexpressed in multiple malignancies, including SCCs. Furthermore, we demonstrated that Dsg2 activates the MAPK, PI3K/Akt and JAK/Stat3 pathways [8]. The mechanism by which Dsg2 activates mitogenic signaling is not fully determined but has been speculated to be through EGFR. To determine whether the interaction between Dsg2 and EGFR is relevant to skin cancer development, SCC tissues were immunostained for Dsg2 and EGFR, showing not only upregulation, but also co-localization, of Dsg2 and EGFR in these tissues *in vivo* (Figure 1). Next, to determine whether Dsg2 modulates EGFR, we generated stable HaCaT (spontaneously transformed immortalized keratinocyte) cell lines expressing a short hairpin RNA (shRNA) directed against human Dsg2 (shDsg2) and Green Fluorescent Protein (shGFP) as a negative control. Immunofluorescence (Figure 2A) and immunoblotting (Figure 2B) show reduced expression of Dsg2 protein in HaCaT-shDsg2 knockdown (KD) compared to HaCaT-shGFP. Quantification of the Western blots demonstrate that the shRNA reduced Dsg2 by ~70% and EGFR by ~40% in HaCaT-shDsg2 as compared to control cells (Figure 2B). Collectively, our data demonstrate that knockdown of Dsg2 reduced EGFR level in HaCaT cells. Changes in Dsg2 did not affect the expression of other desmosome-associated proteins in HaCaT cells except desmocollin 2 (Dsc2) (Figure 2C). This result contrasts colon cancer cells [17], where KD of Dsg2 in malignant colonic epithelial cells led to a concomitant increase in Dsc2. The mechanism by which Dsg2/Dsc2 modulates the expression of each other in keratinocytes likely differs from that of simple colon epithelial cells.

**Figure 3:**
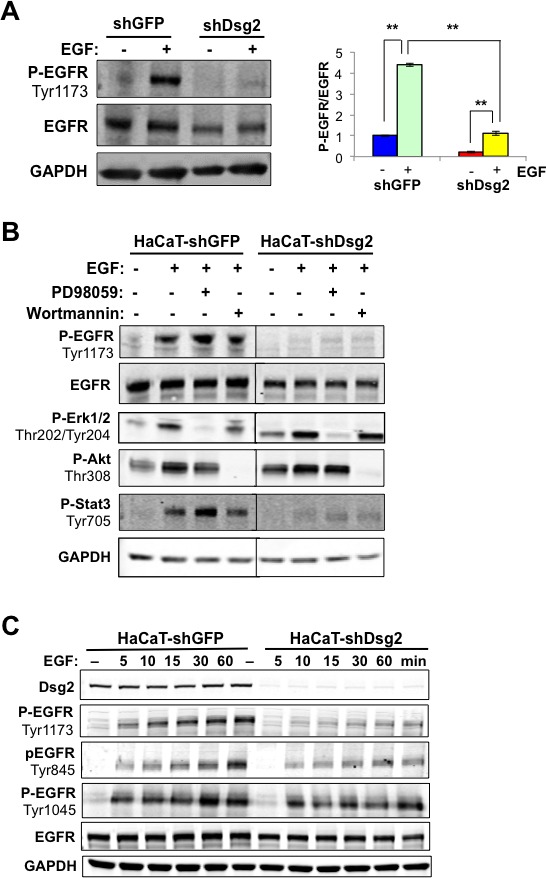
Dsg2 modulates EGFR and Stat3 activation **A.** HaCaT-shGFP and -shDsg2 cells were serum-starved and then stimulated with EGF (10 nM). Total proteins were immunoblotted for active P-EGFR (Tyr1173), EGFR, and GAPDH as loading control. Densitometry was performed, and histogram bars represent the relative amount of Dsg2 and EGFR normalized to GAPDH in untreated cells, and the ratio of P-EGFR (Tyr 1173) to total EGFR after 1 hr of stimulation was quantified and plotted. Data are expressed as average value ± s.e.m of at least 3 independent experiments. Dsg2 (shGFP, 1.00±0.12; shDsg2, 0.25±0.06); EGFR (shGFP, 1.00±0.20; shDsg2, 0.58±0.09); (shGFP, 1.00±0.04; shGFP+EGF, 4.39±0.08; shDsg2, 0.22±0.04; shDsg2+EGF, 1.12±0.11); **p < 0.01; ***p < 0.001; *t*-test. **B.** HaCaT-shGFP and -shDsg2 cells were incubated with PD098059 (50 μM) or Wortmannin (100 nM) for 1 h prior to treatment with EGF (10 nM) for 1 h. Cell lysate was subjected to Western blotting analysis for P-EGFR (Tyr1173), EGFR, P-Erk (Thr202/Tyr204), P-Akt (Thr308), P-Stat3 (Tyr705), and GAPDH for loading control. Results shown are representative of three independent experiments. **C.** HaCaT-shGFP and -shDsg2 cells were treated with EGF (10 nM) for 5, 10, 15, 30 and 60 min. Cells were lysed and total proteins immunoblotted for Dsg2, EGFR, P-EGFR (Tyr1173, Tyr1045 and Tyr845), and GAPDH as loading control.

Next we sought to determine the effect of Dsg2 on EGFR activation. In response to EGF ligand stimulation, control HaCaT-shGFP cells showed a robust increase in phosphorylated EGFR (P-EGFR, Tyr1173), which was dramatically abrogated in Dsg2 KD cells (Figure 3A). Phosphorylation of EGFR at Tyr1173 is critical for downstream MAP kinase signaling [36]. To assess the effect of Dsg2 on the MEK/Erk1/2, PI3K/Akt and JAK/Stat3 signaling pathways, HaCaT-shGFP and -shDsg2 cells were stimulated with EGF and immunoblotted for Phospho-Erk1/2, -Akt, and -Stat3. In response to EGF, activation of EGFR resulted in Erk1/2, Akt and Stat3 phosphorylation (Figure 3B). Reduced expression of Dsg2 did not affect either Erk1/2 or Akt phosphorylation, but dramatically reduced Stat3 phosphorylation (Figure 3B). Treatment with the MEK inhibitor PD98059 or the PI3K inhibitor Wortmannin blocked Erk1/2 and Akt signaling, respectively (Figure 3B). Since EGFR activation is upstream of Erk1/2 and Akt, PD98059 and Wortmannin did not affect EGFR phosphorylation in response to EGF ligand stimulation. Furthermore, Wortmannin had no effect on Stat3 phosphorylation while PD98059 treatment slightly increased Stat3 activation, likely due to blocking the inhibitory Erk1/2-mediated phosphorylation of Stat3 (Ser727) [37].

In spite of reduced phosphorylation of EGFR at tyrosine 1173, Erk1/2 was still activated in response to EGF stimulation. To further assess whether Dsg2 modulates unique EGFR phosphorylation sites, HaCaT-shGFP and -shDsg2 cells were treated with EGF for 5 to 60 min, and protein lysates were immunblotted for P-EGFR at Tyr1173, Tyr1045 and Tyr845 (Figure 3C). These phosphorylation sites are associated with downstream MAPK activation (Tyr1173), c-Cbl-mediated receptor degradation (Tyr1045), and c-Src activation (Tyr845) [38–40]. The results showed that Dsg2 KD reduced EGFR phosphorylation at Tyr1173 and Tyr845 for all time points. Interestingly, phosphorylation at Tyr1045 was immediate—within 5 min after EGF stimulation—and Dsg2 KD only slightly attenuated the signal, suggesting that ubiquitin-mediated receptor degradation is largely unaffected by loss of Dsg2. These results demonstrate that Dsg2 had a distinct role in modulating the phosphorylation of EGFR at Tyr1173 and Tyr845. Furthermore, the MEK/Erk1/2 pathway was activated either independent of EGFR or through a phosphorylation site, different from Tyr1173 and Tyr845 that was not assessed.

**Figure 4:**
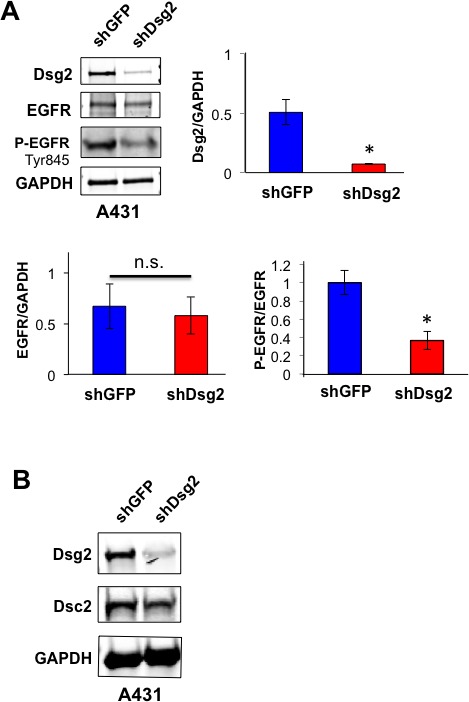
Knockdown of Dsg2 in A431 SCC cells reduces EGFR phosphorylation **A.** A431 cells were stably transfected with shRNA to GFP (shGFP) or Dsg2 (shDsg2) and selected in puromycin. Total cell lysates were immunoblotted for Dsg2, EGFR and P-EGR (Tyr1173). Blotting for GAPDH showed equal loading. Densitometry was performed and bars represent the ratio of Dsg2 to GAPDH, total EGFR to GAPDH and P-EGFR to total EGFR. Data are expressed as average value ± standard error of the mean of three independent experiments. ^n.s.^*p* > 0.05; *p < 0.05; *t*-test. **B.** Immunoblotting of A431-shGFP and -shDsg2 cells for Dsg2 and Dsc2 with GAPDH as loading control. *N* = 3.

In addition to HaCaT cells, we also generated A431 epidermoid cancer cells expressing the shGFP and shDsg2 constructs. A431-shDsg2 cells showed a slight, but not statistically significant decrease in total EGFR (Figure 4A). We attribute this to the substantially high expression of endogenous EGFR in A431 cells [41]. Similar to previous reports, we observed high levels of activated EGFR in control A431 cells [42]. While total EGFR was relatively unchanged, P-EGFR was significantly reduced in A431-shDsg2 cells suggesting that, similar to HaCaT cells, reduced Dsg2 expression suppresses EGFR phosphorylation and activation (Figure 4A). Additionally, similar to the HaCaT-shDsg2 cells, a decrease in Dsc2 expression was observed in A431-shDsg2 cells, further illustrating a lineage-specific modulation of Dsg2/Dsc2 expression (Figure 4B). Due to the high level of endogenous EGFR and P-EGFR in A431 cells, and that the A431 cells would rapidly select against loss of Dsg2, we chose to use HaCaT cells for further mitogenic signaling analysis.

### Dsg2 modulates c-Src phosphorylation and activity

**Figure 5:**
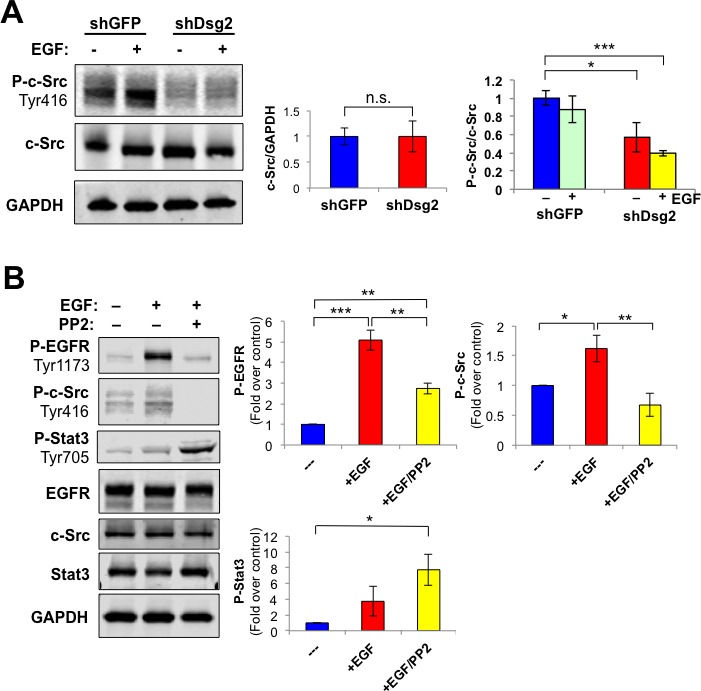
Dsg2 modulates EGFR activation through a c-Src-dependent pathway **A.** HaCaT-shGFP and -shDsg2 cells were stimulated with EGF (10 nM) and proteins immunoblotted for P-c-Src (Tyr416), total c-Src and GAPDH as loading control. Bar graphs show relative ratio of total c-Src/GAPDH (left) and P-c-Src (Tyr416)/total c-Src (right). Data are expressed as average value ± s.e.m. of three independent experiments. c-Src (shGFP, 1.00±0.16; shDsg2, 1.00±0.30); P-c-Src (shGFP, 1.00±0.08; shGFP+EGF, 0.88±0.15); P-c-Src (shDsg2, 0.57±0.16; shDsg2+EGF, 0.40±0.03); Not significant ^n.s.^*p* > 0.05; *p < 0.05; ***p < 0.001; *t*-test. **B.** HaCaT cells were treated with the c-Src inhibitor PP2 (30 μM) for 1 h and then stimulated with EGF (10 nM) for 1 h prior to cell lysis. Total cellular proteins were subjected to immunoblotting for P-EGFR (Tyr1173), EGFR, P-Stat3 (Tyr705), Stat3, P-c-Src (Tyr416), c-Src and GAPDH. Data are expressed as average value ± s.e.m. of six independent experiments. P-EGFR (shGFP, 1.00±0; shGFP+EGF, 5.09±0.49; shGFP+EGF+PP2, 2.74±0.26); P-c-Src (shGFP, 1.00±0; shGFP+EGF, 1.62±0.22; shGFP+EGF+PP2, 0.67±0.20); P-Stat3 (shGFP, 1.00±0; shGFP+EGF, 3.74±1.85; shGFP+EGF+PP2, 7.75±1.99); Not significant ^n.s.^*p* > 0.05; *p < 0.05; **p < 0.01; ***p < 0.001; *t*-test.

The proto-oncogene c-Src is a known regulator and effector of EGFR and Stat3 activation, a transcription factor with oncogenic potential and anti-apoptotic activities [43–45]. In order to determine whether the effect of Dsg2 on EGFR is mediated through c-Src, we assessed the levels of total and active phosphorylated c-Src. Consistent with previous findings, we observed constitutively active P-c-Src (Tyr416) in control HaCaT-shGFP cells (Figure 5A) [46]. Dsg2 did not affect total c-Src; however, activated P-c-Src (Tyr416) was dramatically reduced in the Dsg2 KD cells (Figure 5A). Inhibition of c-Src with the inhibitor PP2 partially abrogated phosphorylation of EGFR in response to EGF ligand in HaCaT cells (Figure 5B), confirming previous findings that c-Src acts both upstream as well as downstream of EGFR [47]. Thus, the Dsg2-dependent EGFR activation may be modulated, in part, by c-Src. Interestingly, inhibition of c-Src slightly increased Stat3 activation (Figure 5B). Reciprocal regulation of c-Src and Stat3 activation has been observed in non-small cell lung cancer cell lines (NSCLC) or tumor xenografts treated with anti-c-Src modalities and in NSCLC human patients [48].

### Dsg2 alters composition of lipid rafts and activates c-Src and EGFR

**Figure 6:**
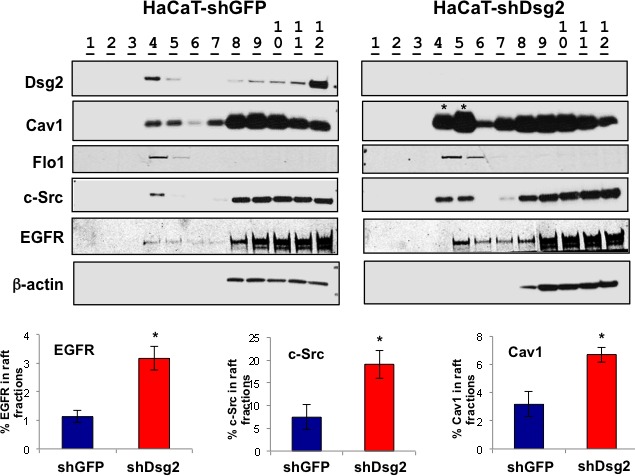
EGFR and c-Src signaling is mediated, in part, through lipid rafts HaCaT-shGFP and -shDsg2 cells were lysed in 1% TX-100 TNE lysis buffer and proteins separated by ultracentrifugation (38,000 RPM) over a sucrose gradient (5, 35, and 45%). Twelve 1 mL fractions were collected from the top and prepared for immunoblot analysis using antibodies specific for Dsg2, Cav1, flotillin 1 (Flo1), c-Src, EGFR and Actin. Light-density lipid raft fractions: 4 and 5. Bands were quantitated and bar graphs show relative ratio of fractions 4 and 5 to all fractions. Loss of Dsg2 increases EGFR, c-Src, and Cav1 in the lipid raft fractions. EGFR (shGFP, 1.13±0.21; shDsg2, 3.17±0.41); c-Src (shGFP, 7.46±2.68; shDsg2, 19.11±3.08); Cav1 (shGFP, 3.17±0.88; shDsg2, 6.70±0.54); *p < 0.05; *t*-test.

Our previous work identified an interaction between Dsg2 and caveolin 1 (Cav1), a known negative regulator of the activities of c-Src and EGFR [22, 49, 50]. However, it is not known whether Dsg2 modulates EGFR and c-Src activity through lipid rafts. Down-regulation of Dsg2 in HaCaT keratinocytes did not alter total Cav1 level (not shown). To determine whether Dsg2 modulates Cav1 membrane raft localization, we relied on the fact that caveolae are buoyant Triton X-100 insoluble membrane fractions and can be isolated through sucrose density gradient centrifugation [22]. In control cells, we detected Cav1, flotillin 1, c-Src and, to a lesser level, EGFR in the light density raft fractions 4 and 5 that are demarcated as containing constituents of caveolae, per the immunoblot for Cav1 (Figure 6). Reduced expression of Dsg2 shifted a higher portion of total Cav1, c-Src and EGFR into the raft fractions. In contrast, lipid raft localization of flotillin-1, a scaffolding protein associated with planar-type lipid rafts, was not affected by Dsg2 expression. Cav1 negatively regulates cellular signaling by sequestering signaling molecules in their inactive state within the caveolae [25]. Together these data suggest that Dsg2 enhances activation of c-Src and EGFR by disrupting their association with lipid rafts.

**Figure 7:**
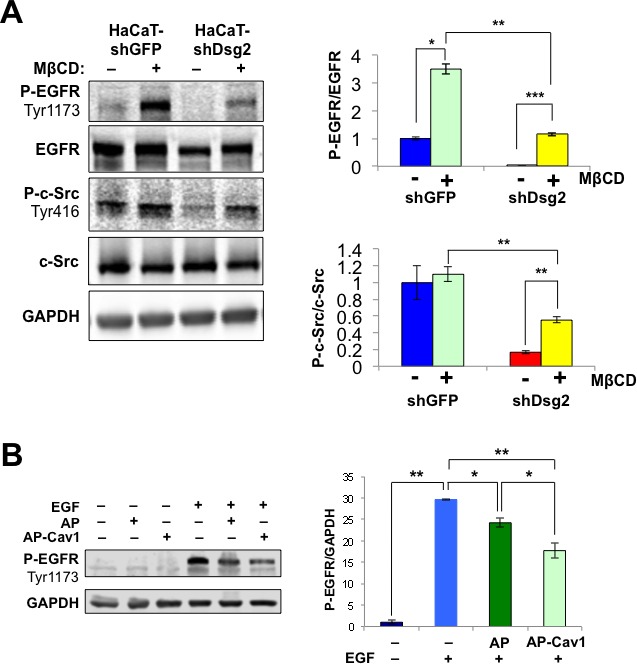
EGFR signaling is mediated through Cav1 and lipid rafts **A.** HaCaT-shDsg2 and -shGFP cells were treated with 1% MβCD for 1 h and total protein lysates were immunoblotted for P-EGFR (Tyr1173), EGFR, P-c-Src (Tyr416), c-Src and GAPDH. Bar graphs show relative ratio of P-EGFR to total EGFR and P-c-Src to total c-Src. Data are expressed as average value ± s.e.m. of three independent experiments. P-EGFR (shGFP, 1.00±0.16; shGFP+MβCD, 2.64±0.12; shDsg2, 0.04±0.01; shDsg2+MβCD, 1.07±0.04); P-c-Src (shGFP, 1.00±0.20; shGFP+MβCD, 1.10±0.09; shDsg2, 0.17±0.02; shDsg2+MβCD, 0.55±0.04); ^n.s.^*p* > 0.05; * *p* < 0.05; *t*-test. **B.** HaCaT cells were incubated with the biotinylated AP ([(biotin)-RQPKIWFPNRRKPWKK-(OH)]; 5 μM) or the Cav-1 consensus binding peptide conjugated to AP (AP-Cav1; [(biotin)-RQPKIWFPNRRKPWKKDGIWKASFTTFTVTKYWFYR-(OH)]; 5 μM) for 1 h prior to stimulation with 10 ng/mL EGF for 1 h. Total cell lysates were immunoblotted for P-EGFR (Tyr1173) and GAPDH (for equal loading). Bar graph shows quantitated values from 3 independent experiments.

Lipid raft-mediated internalization potentially serves as a mechanism for EGFR degradation, independent from clathrin-dependent endocytosis and membrane recycling. Given that lipid rafts can repress extended EGFR activation and loss of Dsg2 attenuates receptor activation, disruption of either the EGFR-lipid raft or lipid raft-Dsg2 interactions should promote receptor activation. Methyl-β-cyclodextrin (MβCD) perturbs lipid raft structure and releases its constituents by chelating cholesterol away from the rafts. Treatment of HaCaT-shGFP and HaCaT-shDsg2 cells with MβCD did not alter total level of c-Src or EGFR, but increased their activation (Figure 7A). Interestingly, KD of Dsg2 decreased the activation of P-c-Src and P-EGFR in response to MβCD, corroborating with the observed decrease in total EGFR expression in HaCaT-shDsg2 cells. To further demonstrate that EGFR activation is mediated through Cav1 and caveolae, we utilized a fusion of the cell permeable *Drosophila* Antennapedia homeodomain and the Cav1 scaffolding domain (Cav1-AP) or a non-specific peptide as a control (AP). This Cav1-AP peptide would disrupt the interaction between Cav1 and its binding partners including, Dsg2 and EGFR [20]. In unstimulated HaCaT cells, AP or AP-Cav1 peptides did not have an effect on EGFR phosphorylation (Figure 7B). EGFR phosphorylation increased in response to EGF ligand stimulation and while the AP control peptide impaired EGFR phosphorylation, AP-Cav1 significantly reduced the phosphorylation level (Figure 7B). We previously showed that AP-Cav1, but not AP, slightly reduced Dsg2 level in lipid raft fractions [22]. Interestingly, AP-Cav1 had no effect on the activation of EGFR in HaCaT-shDsg2 cells (not shown), which already had abrogated ligand-induced EGFR activation, further demonstrating that connection between receptor activation and Dsg2. Both MβCD and AP-Cav1 treatment in Dsg2-depleted cells illustrate that EGFR activation in keratinocytes can be dependent upon the ability of Dsg2 to modulate receptor association with caveolae.

**Figure 8:**
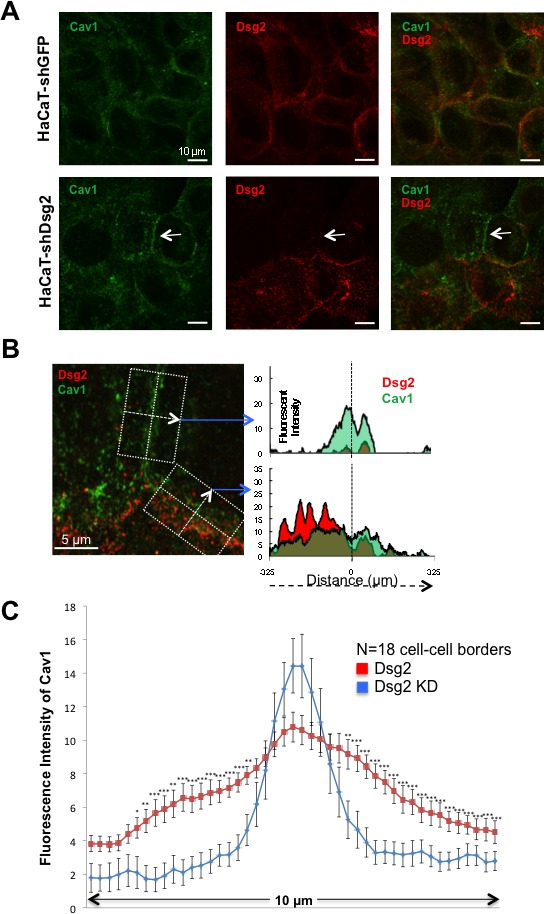
Dsg2 displaces Cav1 from cell-cell borders **A.** Immunofluorescent staining of HaCaT-shGFP and HaCaT-shDsg2 cells for Cav1 (green), Dsg2 (red) and visualized by confocal microscopy showing more defined Cav1 staining in the absence of Dsg2 (arrow). **B.** Staining was visualized and imaged by STED super-resolution microscopy. The average intensity of Cav1 and Dsg2 staining was calculated across the cell-cell border of the boxed areas of the merged STED image. Briefly, measurements of the fluorescent intensity for both Cav1 and Dsg2 was obtained in ImageJ with a line 6.25 μm long centered on the Cav1 staining (dashed vertical line) and originating from left to right (dashed arrow) perpendicularly across the width of the cell-cell border. Multiple line measurements were taken along the length of the distinct cell-cell borders and intensity values averaged to produce both the Dsg2-absent (top graph) and Dsg2-positive cell border staining of Cav1 and Dsg2. Scale bars = 5 μm. **C.** Average fluorescent intensity quantitated from Dsg2 (red) and Dsg2 KD (blue) cell-cell borders (n = 18 each). *p < 0.05, **p < 0.01***p < 0.001; *t*-test.

To more precisely illustrate that expression of Dsg2 disrupts Cav1 membrane localization, we utilized stimulated emission depletion (STED) super-resolution microscopy [51]. We relied on the fact that the knockdown of Dsg2 was not complete in the HaCaT-shDsg2 cells, with some patches of HaCaTs expressing Dsg2 and permitting the visualization of Cav1 in both the presence and absence of Dsg2 (Figure 8A). Confocal images of Dsg2 (red), Cav1 (green) and merged demonstrate the presence of Dsg2 and Cav1 at cell-cell contacts in both Dsg2-expressing and Dsg2-KD HaCaTs (Figure 8A). Utilizing STED at an intersection of three cell-cell borders for enhanced analysis, Cav1 signal was concentrated at the cell-cell border between cells lacking Dsg2 but became dispersed and scattered in the presence of Dsg2 (Figure 8B). This suggests that the physical presence of Dsg2 disrupted well-defined Cav1 localization to the cell membrane. By calculating the intensity of Dsg2 and Cav1 staining perpendicularly across both Dsg2-expressing and non-expressing cell-cell borders, it was apparent that higher Dsg2 expression redistributed Cav1 intracellularly (Figure 8B). This disruption of Cav1 by Dsg2 was further confirmed by calculating the relative fluorescent intensity of Cav1 at cell contacts in Dsg2-overexpressing and Dsg2-KD HaCaTs from confocal images (Figure 8C). HaCaTs without cell-cell Dsg2 staining had a distinct peak of Cav1 centered in and immediately around the cell membrane whereas Dsg2-expressing cells generally had Cav1 distributed further into the cytosol. These results begin to suggest that Dsg2 may promote Cav1/caveolae internalization; indeed, the cytosolic shift of Cav1 was even apparent in Dsg2-expressing cells that bordered cells without Dsg2 (data not shown). We observed modulated levels of Cav1 in the perimembrane region, suggesting an important dynamic between Dsg2 and membrane-presentation of Cav1.

### Dsg2 enhances SCC cell proliferation and migration through EGFR and c-Src

**Figure 9:**
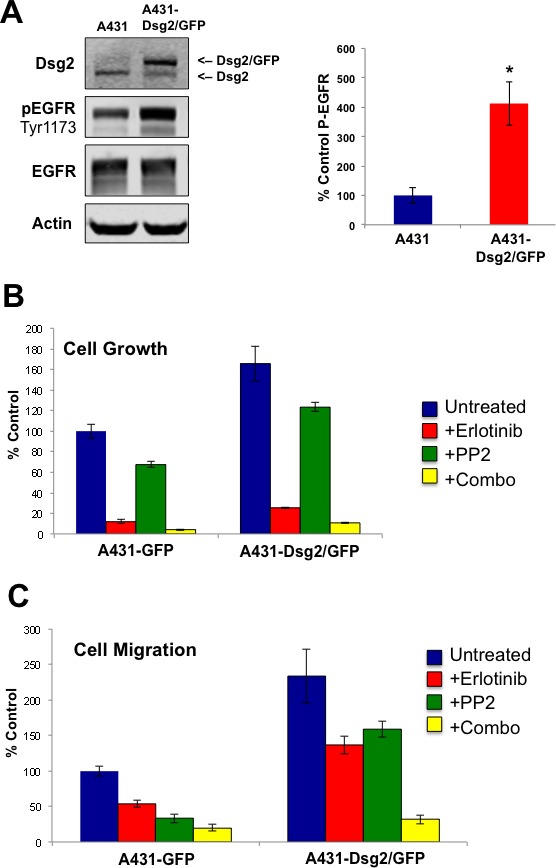
Dsg2-mediated SCC cell growth and migration is dependent on EGFR and c-Src **A.** A431 cells were transduced with retroviruses encoding for GFP-labeled Dsg2. Total protein lysates from A431 and A431-Dsg2/GFP cells were immunoblotted for Dsg2, pEGFR, and EGFR. Actin served as loading control. P-EGFR (A431, 100±26.5; A431-Dsg2/GFP, 413±74.5) *p < 0.05; ***p < 0.001; *t*-test. The effect of Dsg2 on cell proliferation and migration was assessed. **B.** A431 and A431-Dsg2/GFP cells were plated at low density for 6 days in the presence of Erlotinib, PP2 or both. Cells were trypsinized and counted (*n* = 14). A431, 100.0±6.6; A431+Erlotinib, 11.9±1.9; A431+PP2, 67.4±3.0; A431+Erlobtinib+PP2, 4.1±0.7; A431-Dsg2/GFP, 165.7±16.8; A431-Dsg2/GFP+Erlotinib, 25.4±0.9; A431-Dsg2/GFP+PP2, 123.4±4.5; A431-Dsg2/GFP+Erlobtinib+PP2, 10.7±0.8. **C.** For migration, cells treated with Erlotinib, PP2 or both in serum free medium were plated in the top chamber and allowed to migrate through an uncoated Transwell membrane in response to serum-containing medium in the lower chamber. The membranes were fixed and stained with methylene blue. Cells were counted and presented as percentages of the control migration (n = 9). A431, 100.0±7.1; A431+Erlotinib, 53.7±4.6; A431+PP2, 33.1±5.9; A431+Erlotinib+PP2, 20.0±4.9; A431-Dsg2/GFP, 233.8±37.6; A431-Dsg2/GFP+Erlotinib, 136.7±12.6; A431-Dsg2/GFP+PP2, 159.1±11.3; A431-Dsg2/GFP+Erlotinib+PP2, 31.9±6.4.

To further study the ability of Dsg2 to modulate the activation of EGFR and its relevance to cancer, we generated stable A431 SCC cells expressing a GFP-labeled Dsg2 (upper band; Figure 9A). We note that the endogenous Dsg2 and the ectopically expressed Dsg2-GFP localized similarly in light density fractions confirming that Dsg2-GFP did not affect endogenous Dsg2 lipid raft association (not shown). We opted to use A431 for additional study, as it is a well-characterized keratinocyte-derived tumorigenic cell line amenable to both Dsg2 overexpression and phenotypic analysis. Furthermore, unlike HaCaTs, A431 cells can be used unstimulated in the transwell migration assay. Interestingly, overexpression of Dsg2-GFP did not significantly alter the level of EGFR but dramatically enhanced endogenous EGFR activation (Figure 9A). Using these cell lines, we next sought to determine the effect of Dsg2 on cancer cell growth and migration and whether it is mediated through EGFR and c-Src. Dsg2 enhanced SCC cell growth, which was dramatically abrogated in the presence of the EGFR inhibitor Erlotinib (Figure 9B). The c-Src inhibitor PP2 only slightly reduced cell growth in response to Dsg2, suggesting the prevalence of upstream EGFR activation as a determinant of growth in these cells. However, the combination of EGFR and c-Src inhibitors was synergistic in reducing cell proliferation (Figure 9B). In addition to modulating growth, Dsg2 also enhanced cell migration through a transwell migration assay in response to Fetal Bovine Serum (FBS) as a chemotactic factor (Figure 9C). The Dsg2-mediated increase in migration was partially inhibited by Erlotinib and PP2 (Figure 9C) further demonstrating the dependence of cell motility and invasion upon EGFR and, to an extent, c-Src activation. The combination of both inhibitors further abrogated the migration of A431-Dsg2/GFP cells. In summary, these results demonstrate that Dsg2 plays an active role in modulating epithelial cell growth and migration through EGFR and c-Src.

### Dsg2 enhances cell growth and migration independent of desmosomes

**Figure 10:**
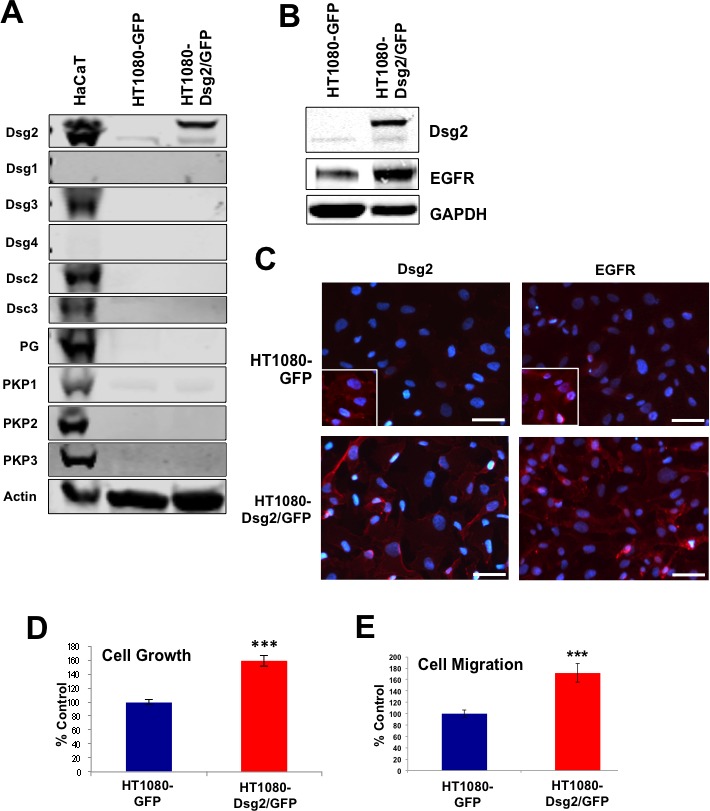
Dsg2 enhances fibrosarcoma cell growth and migration HT1080 cells were transduced with retroviruses encoding for GFP-labeled Dsg2. **A.** Total protein lysates from HaCaT, HT1080 and HT1080-Dsg2/GFP cells were immunoblotted for desmosomal proteins and actin as loading control. **B.** HT1080 and HT1080-Dsg2/GFP cell lysates were immunoblotted for Dsg2 and EGFR showing enhanced EGFR level in response to Dsg2. **C.** Immunofluorescence of HT1080 and HT1080-Dsg2/GFP cells for Dsg2 and Cav1. Nuclei stained blue with DAPI. Bar = 100 μm. **D.** Proliferation was assessed by cell counting. **E.** Migration potential was assessed by transwell migration assay with HT1080 and HT1080-Dsg2/GFP cells. Migrated cells were counted and presented as percentages of the control.

HaCaT and A431 epithelial cells express desmosomal proteins and establish desmosomal contacts. Desmogleins, including Dsg2, are incorporated into these cell-cell junctions, posing a challenge to delineate whether the desmosome-bound or desmosome-free Dsg2 exerted the observed effects on growth and migration. To assess the role of Dsg2 independent of desmosomes, we employed the HT1080 fibrosarcoma-derived cells. These cells express low levels of endogenous Dsg2, but no significant amounts of other desmosomal proteins (Figure 10A). Stable HT1080 cell lines were established expressing Dsg2-GFP; Western blotting analysis confirmed expression of the GFP-tagged Dsg2 protein (upper band; Figure 10B). Immunofluorescence showed both high membrane and cytoplasmic localization of Dsg2 (Figure 10C). Furthermore, Dsg2 dramatically enhanced EGFR levels (Figure 10B and 10C). Similar to that observed in A431 SCC cells, ectopic overexpression of Dsg2 enhanced HT1080 cell proliferation (Figure 10D) and migration (Figure 10E). These results support the non-desmosome role of Dsg2 in cell proliferation and migration, possibly through regulating EGFR.

## DISCUSSION

In this study we have provided evidence that reduced expression of the desmosomal junction protein Dsg2 in epithelial keratinocytes reduces the activation of both EGFR and c-Src, leading to a reduction in cell proliferation. We also have demonstrated that Dsg2 displaces Cav1, the major integral membrane protein of caveolae, as well as c-Src and EGFR, from lipid rafts (Figure 6). In a similar manner, disruption of lipid rafts with the cholesterol-chelating agent MβCD shifts EGFR and c-Src out of lipid raft fractions, resulting in enhanced phosphorylation and activation of these two important signaling components. Additionally, overexpression of Dsg2 potently activates EGFR and enhances proliferation and migration tumorigenic A431 and HT1080 cells. Taken together, these data suggest a crosstalk between cell adhesion and mitogenic signaling and that Dsg2 utilizes lipid rafts as a platform to facilitate the activation of EGFR and c-Src signaling.

EGFR phosphorylation at Tyr845 has been shown to be c-Src-dependent, which, in turn, activates Stat3 transcriptional activity [39]. In addition to the role of Tyr1173 phosphorylation in downstream MAPK activation, SHP-1, a protein tyrosine phosphatase, associates with activated EGFR at that residue and attenuates receptor-mediated downstream signaling. While it is not possible to rule out the effect of Dsg2 downregulation on other signal transduction pathways that alter MAPK pathway activation, decreased EGFR Tyr1173 phosphorylation has been shown to interfere with the recruitment of SHP-1, but not SHC/Grb2 (mediators of EGFR-induced MAPK activation), to active EGFR [40]. Without the inhibitory phosphatase activity of SHP-1, decreased EGFR activation mediated by loss of Dsg2 may not necessarily lead to significantly decreased MAPK signaling. Indeed, the most profound effect observed on downstream mitogenic signaling factors as a result of decreased receptor activation from Dsg2 knockdown was on the c-Src/Stat3 signaling axis.

Cell adhesion proteins are emerging as key players in cancer progression and metastasis. We previously showed that the desmosomal cadherin Dsg2 is highly upregulated in several skin malignancies [9]. Furthermore, overexpression of Dsg2 in the epidermis of transgenic mice enhances EGFR level and activates mitogenic signaling leading to epidermal hyperplasia and sensitivity to tumor development [8]. EGFR is overexpressed and/or activated in many human tumors including SCCs and is often correlated to tumor aggressiveness [52]. Indeed, we observed consistent co-localization of elevated Dsg2 and EGFR expression in human SCC samples (Figure 1) suggesting a functional interaction in the disease. In cultured keratinocytes, overexpression of EGFR enhances cell proliferation and survival [53, 54]. Overexpression of Dsg2 induces potent EGFR expression and activation that stimulates cell proliferation and migration (Figures 9 and 10). This effect is not desmosomal-dependent, as the results were recapitulated in the fibrosarcoma-derived HT1080 cell line that does not express desmosomes. Interestingly, the HT1080 cell line expressed a small amount of endogenous Dsg2 that, having been observed previously, further suggests a desmosome-independent role for Dsg2 [55]. Thus, our finding here that Dsg2 can modulate EGFR activation is a critical link that connects cell-cell adhesion to mitogenic signaling in skin cancer development. Dsg2 depletion in SK-CO15 colon cancer cells also disrupts EGFR signaling [56]. However unlike HaCaT keratinocytes, loss of Dsg2 does not alter the total level of EGFR in SK-CO15 colon cancer cells. This may be due to the constitutively high level of EGFR in cancers cells such as A431 or SK-CO15.

In breast cancer cells, lipid rafts provide a platform for the interaction of EGFR and c-Src, leading to activation of cellular survival signaling [57]. Here, we observed that reduced expression of Dsg2 decreases active P-c-Src, which can regulate EGFR activation [43]. We propose that by altering Cav1 composition in lipid rafts, Cav1 has decreased capacity for sequestering and negatively regulating signaling complexes within caveolae. Indeed, in the presence of Dsg2, the level of c-Src and EGFR is similarly reduced in lipid raft fractions.

Upon ligand binding, activated receptor tyrosine kinases such as EGFR undergo rapid endocytosis, internalization and sorting to lysosomes for degradation [58]. It is generally accepted that clathrin-mediated endocytosis is the major pathway for internalization of EGFR [59, 60]. Mounting evidence, however, supports the role of membrane lipid rafts in modulating EGFR level and activation. Treatment of HaCaT keratinocytes with MβCD leads to accumulation of EGFR in large clusters outside of the disrupted rafts resulting in enhanced tyrosine kinase activity possibly due receptor clustering or loss of inhibition [61]. It has been proposed that autoactivation of EGFR may lead to internalization and targeting for degradation through lipid rafts. Indeed, the absence of Dsg2 has an effect both on the localization of EGFR to lipid rafts (Figure 6) and the distribution of Cav1 around the membrane (Figure 8); interrogating the precise mechanism that Dsg2 influences EGFR trafficking through lipid rafts will require additional study. In a manner similar to manipulating lipid rafts with cholesterol chelating agents, displacement of components from lipid rafts results in activation of numerous signaling cascades as well as alteration of differentiation markers such as involucrin [62]. Transcriptional profiling of keratinocytes after treatment with MβCD and identified over 3,000 differentially regulated genes [63]. It is evident that altering lipid raft composition has a significant impact on cellular communication and epithelial homeostasis. Importantly, our findings provide a potential mechanism for keratinocyte morphogenesis and malignant transformation by Dsg2.

A number of targeted therapies directed against both EGFR and c-Src are approved for the usage in a variety of malignancies; no therapies currently exist for Cav1 [64]. EGFR-targeting small-molecule (gefitinib, erlotinib, lapatinib, etc.) and antibody-based (cetuximab and panitumumab) treatment modalities have been developed to either block ligand-dependent receptor activation or cytoplasmic kinase activity. EGFR inhibition-based therapies are currently in use for a wide variety of solid malignancies including non-small cell lung cancer, breast cancer, and prostate cancer [65–67]. Though effective in naïve disease, patients often experienced severe side effects from the treatments and, depending on the malignancy, progress to an EGFR-insensitive disease. Additionally, a number of small-molecule tyrosine kinase inhibitors (TKI) of c-Src have been shown to have significant clinical effect, with the most well characterized inhibitor being dasatanib. Dasatanib has been used extensively as a second-line therapy in patients with chronic myelogenous leukemia with the BCR-ABL fusion protein, but, along with other c-Src-targeting TKIs, has produced generally disappointing results in solid malignancy clinical trials [68]. Combinatorial strategies utilizing both EGFR and c-Src-directed inhibitors are being explored in various solid tumors. Neither EGFR nor c-Src-targeted therapies are approved for usage in cutaneous SCCs, though clinical observations and trials with cetuximab and gefitinib monotherapy have shown efficacy in treating unresectable disease [69–71]. Given the overexpression of EGFR and Dsg2 observed in SCCs, and the relatively low expression of Dsg2 in the normal adult interfollicular epidermis, a combinatorial therapy of EGFR inhibitors with a Dsg2-directed modality may prove useful in enhancing the effect of EGFR inhibition while simultaneous limiting the adverse reactions to the treatment [69].

Taken together, the data obtained here suggest that Dsg2 may play a significant role in tumor development by positively regulating EGFR level and signaling through a c-Src and Cav1 dependent manner.

## MATERIALS AND METHODS

### Antibodies

Antibodies used were: H145 Dsg2 (1:10,000), Cav1 (1:40,000), and GAPDH (1:3,000; Santa Cruz, Santa Cruz, CA); Actin (1:100,000; Calbiochem, Billerica, MA);; Flotillin 1, c-Src, P-c-Src Tyr416, P-c-Src Tyr527, EGFR, P-EGFR Tyr1173, P-EGFR Tyr1045, P-EGFR Tyr845, P-Erk1/2 (Thr202/Tyr204), P-AKT (Thr308), P-Stat3 (Tyr705) (1:1,000; Cell Signaling, Danvers, MA); 10D2 Dsg2 (1:2), 27B2 Dsg1 (1:100), 5H10 Dsg3 (1:100), 18D4 Dsg4 (1:100), desmocollin-2/3 7G6 (1:10,000), 20F6 desmoplakin (1:50), 14B11 plakophillin-1 (1:50), 8H6 plakophillin-2 (1:50), 19A5 plakophillin-3 (1:100), 11E4 plakoglobin (1:100), 4A2 E-cadherin (1:2,500), 6F9 β-catenin (1:1,000) [72]; Secondary antibodies: Alexa Fluor-488 & −594 (1:400, Molecular Probes, Eugene, OR); HRP (1:5,000; Jackson Labs, Bar Harbor, ME); IRDye 680 & 800(1:20,000; LI-COR Biosciences, Lincoln, NE); Secondary antibodies for STED-imaging: Mega520-conjugated anti-Rabbit (Sigma-Aldrich) and Star635P-conjugated anti-Mouse (Abberior GmbH, Göttingen, Germany).

### Molecular constructs

Short hairpin RNAs (shRNA) targeting human Dsg2 were generated by the synthesis of oligonucleotides per the pSuper retro puro user manual (Oligoengine, Seattle, WA) using the gene specific sequences: (‘5-GAT CCC CGA GAG GAT CTG TCC AAG AAT TCA AGA GAT TCT TGG ACA GAT CCT CTC TTT TT-3′ and ‘5-AGC TTA AAA AGA GAG GAT CTG TCC AAG AAT CTC TTG AAT TCT TGG ACA GAT CCT CTC GGG-3′). Control shRNA targeting Green Fluorescent Protein was generated as previously described [73]. Oligos were annealed and ligated to pSuper-retro-puro. Retroviral particles were generated and stable HaCaT (immortal keratinocytes) and A431 (squamous carcinoma) cell lines were selected in medium containing 2 μg/mL puromycin. The Dsg2 cDNA was subcloned upstream of GFP in pEGFP-N1 (Clontech, Mountainview, CA). The GFP and Dsg2-GFP cDNAs were subcloned into the retroviral expression vector LZRS-ms-neo and transfected into Phoenix cells. Retroviral particles were generated and stable A431 and HT1080 (fibrosarcoma) cells were selected in G418 (50 μg/ml) as previously described [73]. During the course of this study we made several interesting observations. First, it was difficult to maintain the Dsg2 knock-down phenotype in A431 SCC cells as the cultures would select for Dsg2-expressing cells over time, even in the presence of selection medium. Second, the Dsg2-GFP construct used to overexpress Dsg2 in the A431s did not adequately overexpress the protein in HaCaTs. These cells would often slightly downregulate endogenous Dsg2 and have a similar level of total Dsg2 to that of the control cells.

### Cell culture and drug treatment

HaCaT, A431, and HT1080 cells were maintained in DMEM complete medium containing 10% fetal bovine serum (FBS; Fisher, Waltham, MA) and 1X penicillin/streptomycin (Fisher) as previously described [74, 75]. Cells were incubated in serum-free DMEM for 1 h prior to treatment with EGF (10 ng/mL; Invitrogen, Carlsbad, CA) for the indicated time (0-60 min). In some experiments, cells were pre treated with PD098059 (50 μM; BioMol Research, Plymouth Meeting, PA), Wortmannin (100 nM; BioMol Research), or PP2 (10 μM; Millipore Corp., Billerica, MA) for 1 h. prior to EGF stimulation. To disrupt lipid rafts, cells were treated with MβCD (1 %) for 1 h.

### Proliferation assay

Cellular proliferation rate was determined by counting the number of cells after 6 days of proliferation. Cells were seeded in triplicate at 5×10^3^ cells per chamber of 12-well culture plates (Corning, Corning, NY) in complete DMEM with DMSO, Erlotinib (1μM), PP2 (5μM) or the combination treatment. Six days post seeding, cells were trypsinized (0.25% Trypsin-EDTA) and counted.

### Transwell migration assay

Cell migration was performed with 5×10^3^ HT1080 cells or 5×10^4^ A431 cells plated in the top chamber of the Transwell insert on an uncoated membrane (8 μm pores for A431 and 2 μm pores for HT1080; Corning). Cells were seeded in the upper chamber in serum-free DMEM with the same concentration of inhibitors utilized for the proliferation assay; 10% FBS-containing DMEM was the chemoattractant in the lower chamber. Cells were allowed to migrate for 18-24 h then rinsed in PBS, fixed in paraformaldehyde and stained with 0.5% crystal violet in 50% methanol. Unmigrated cells in the upper chambers were removed with a cotton swab, and migrated cells in the lower chambers were imaged in 5 random fields using an inverted microscope (EVOS, Life Technologies, Grand Island, NY).

### Isolation of lipid raft fractions

Cells were lysed with TNE buffer (25 mM Tris-HCl, pH 7.5, 150 mM NaCl, 5 mM EDTA) containing 1% TX-100 and supplemented with PMSF (1 mM), protease inhibitors (Roche Diagnostics, Indianapolis, IN), and phosphatase inhibitors (Sigma, St. Louis, MO) and homogenized with a Dounce Homogenizer. Equal volume of 90% sucrose and cell lysate were mixed and overlayed with equal volume of 35% sucrose followed by 5% sucrose all in TNE buffer. Samples were centrifuged at 4^°^C for 18-20 h at 38,000 rpm using an SW41Ti rotor (Beckman Coulter, Brea, CA). From top, twelve fractions were collected and prepared for Western blotting analysis.

### Cell immunoblotting and immunohistochemistry

Cells were lysed with lysis buffer (50 mM Tris-HCl pH 7.5, 150 mM NaCl, 5 mM EDTA, and 1% TX-100) supplemented with PMSF, protease and phosphatase inhibitors and heated to 95°C for 10 m in Laemmli buffer. Proteins were resolved over SDS-PAGE gel (Bio-Rad Labs, Hercules, CA). Membranes were blocked in Odyssey blocking buffer (LI-COR, Lincohn, NE) and incubated in primary antibody overnight at 4^°^C, followed by secondary antibody. Infrared bands were visualized by LI-COR Odyssey imaging system. For immunostaining, cultured cells were fixed in 1% paraformaldehyde and permeabilized with 1% TX-100 in PBS. Immunostaining on formalin-fixed paraffin embedded sections was performed as previously described [8].

### Stimulated emission depletion microscopy (STED)

After immunostaining, cells were mounted in Mowiol-DABCO (Sigma) and imaged with a Leica TCS STED (SP5) confocal microscope (Buffalo Grove, IL, USA). Mega520 and Star635P were excited at 531 nm and 635 nm respectively using pulsed diode lasers (PicoQuant, Berlin, Germany). Fluorescent molecules were depleted at 765 nm with a tunable titanium-sapphire laser (MaiTai HP, Spectra- Physics, Santa Clara, CA) and emission was detected with an avalanche photodiode detector (Perkin Elmer, Waltham, MA) at 665-705 nm range. To avoid overlap between the Mega520 and Star635P channels, image acquisitions were sequentially conducted at 531 then 635 nm (Laboratory of Biophysics, Institute of Biomedicine and MediCity Research Laboratories, University of Turku, Finland).

## References

[1] Mahoney MG, Hu Y, Brennan D, Bazzi H, Christiano AM, Wahl JKr (2006). Delineation of diversified desmoglein distribution in stratified squamous epithelia: implications in diseases. Exp Dermatol.

[2] Holthöfer B, Windoffer R, Troyanovsky S, Leube RE (2007). Structure and function of desmosomes. Int Rev Cytol.

[3] Pilichou K, Nava A, Basso C, Beffagna G, Bauce B, Lorenzon A, Frigo G, Vettori A, Valente M, Towbin J, Thiene G, Danieli GA, Rampazzo A (2006). Mutations in desmoglein-2 gene are associated with arrhythmogenic right ventricular cardiomyopathy. Circulation.

[4] Wang H, Li ZY, Liu Y, Persson J, Beyer I, Möller T, Koyuncu D, Drescher MR, Strauss R, Zhang XB, Wahl JKr, Urban N, Drescher C, Hemminki A, Fender P, Lieber A (2011). Desmoglein 2 is a receptor for adenovirus serotypes 3, 7, 11 and 14. Nat Med.

[5] Camargo LM, Zhang XD, Loerch P, Caceres RM, Marine SD, Uva P, Ferrer M, de Rinaldis E, Stone DJ, Majercak J, Ray WJ, Yi-An C, Shearman MS, Mizuguchi K (2015). Pathway-based analysis of genome-wide siRNA screens reveals the regulatory landscape of APP processing. PLoS One.

[6] Kamekura R, Nava P, Feng M, Quiros M, Nishio H, Weber DA, Parkos CA, Nusrat A (2015). Inflammation-induced desmoglein-2 ectodomain shedding compromises the mucosal barrier. Mol Biol Cell.

[7] Eshkind L, Tian Q, Schmidt A, Franke WW, Windoffer R, Leube RE (2002). Loss of desmoglein 2 suggests essential functions for early embryonic development and proliferation of embryonal stem cells. Eur J Cell Biol.

[8] Brennan D, Hu Y, Joubeh S, Choi YW, Whitaker-Menezes D, O'Brien T, Uitto J, Rodeck U, Mahoney MG (2007). Suprabasal Dsg2 expression in transgenic mouse skin confers a hyperproliferative and apoptosis-resistant phenotype to keratinocytes. J Cell Science.

[9] Brennan D, Mahoney MG (2009). Increased expression of Dsg2 in malignant skin carcinomas: A tissue-microarray based study. Cell Adh Migr.

[10] Kurzen H, Munzing I, Hartschuh W (2003). Expression of desmosomal proteins in squamous cell carcinomas of the skin. J Cutan Pathol.

[11] Schäfer S, Koch PJ, Franke WW (1994). Identification of the ubiquitous human desmoglein, Dsg2, and the expression catalogue of the desmoglein subfamily of desmosomal cadherins. Exp Cell Res.

[12] Denning MF, Guy SG, Ellerbroek SM, Norvell SM, Kowalczyk AP, Green KJ (1998). The expression of desmoglein isoforms in cultured human keratinocytes is regulated by calcium, serum, and protein kinase C. Exp Cell Res.

[13] Barber AG, Castillo-Martin M, Bonal DM, Rybicki BA, Christiano AM, Cordon-Cardo C (2014). Characterization of desmoglein expression in the normal prostatic gland. Desmoglein 2 is an independent prognostic factor for aggressive prostate cancer. PLoS One.

[14] Abulrob A, Giuseppin S, Andrade MF, McDermid A, Moreno M, Stanimirovic D (2004). Interactions of EGFR and caveolin-1 in human glioblastoma cells: evidence that tyrosine phosphorylation regulates EGFR association with caveolae. Oncogene.

[15] Teh MT, Parkinson EK, Thurlow JK, Liu F, Fortune F, Wan H (2011). A molecular study of desmosomes identifies a desmoglein isoform switch in head and neck squamous cell carcinoma. J Oral Pathol Med.

[16] Biedermann K, Vogelsang H, Becker I, Plaschke S, Siewert JR, Hofler H, Keller G (2005). Desmoglein 2 is expressed abnormally rather than mutated in familial and sporadic gastric cancer. J Pathol.

[17] Kamekura R, Kolegraff KN, Nava P, Hilgarth RS, Feng M, Parkos CA, Nusrat A (2013). Loss of the desmosomal cadherin desmoglein-2 suppresses colon cancer cell proliferation through EGFR signaling. Oncogene.

[18] Yashiro M, nishioka N, hirakawa K (2006). Decreased expression of the adhesion molecule desmoglein-2 is associated with diffuse-type gastric carcinoma. Eur J Cancer.

[19] Gupta A, Nitoiu D, Brennan-Crispi D, Addya S, Riobo NA, Kelsell DP, Mahoney MG (2015). Cell cycle- and cancer-associated gene networks activated by Dsg2: evidence of cystatin A deregulation and a potential role in cell-cell adhesion. PloS One.

[20] Tong J, Taylor P, Moran MF (2014). Proteomic analysis of the epidermal growth factor receptor (EGFR) interactome and post-translational modifications associated with receptor endocytosis in response to EGF and stress. Mol Cell Proteomics.

[21] Brennan-Crispi DM, Hossain C, Sahu J, Brady M, Riobo NA, Mahoney MG (2015). Crosstalk between Desmoglein 2 and Patched1 accelerates chemical-induced skin tumorigenesis. Oncotarget.

[22] Brennan D, Peltonen S, Dowling A, Medhat W, Green KJ, Wahl JK, Del Galdo F, Mahoney MG (2012). A role for caveolin-1 in desmoglein binding and desmosome dynamics. Oncogene.

[23] Simons K, Toomre D (2000). Lipid rafts and signal transduction. Nat Rev Mol Cell Biol.

[24] Staubach S, Hanisch FG (2011). Lipid rafts: signaling and sorting platforms of cells and their roles in cancer. Expert Rev Proteomics.

[25] Fridolfsson HN, Roth DM, Insel PA, Patel HH (2014). Regulation of intracellular signaling and function by caveolin. FASEB J.

[26] Couet J, Li S, Okamoto T, Ikezu T, Lisanti MP (1997). Identification of peptide and protein ligands for the caveolin-scaffolding domain. Implications for the interaction of caveolin with caveolae-associated proteins. J Biol Chem.

[27] Lee SW, Reimer CL, Oh P, Campbell DB, Schnitzer JE (1998). Tumor cell growth inhibition by caveolin re-expression in human breast cancer cells. Oncogene.

[28] Engelman JA, C.C. W, Yasuhara S, Song KS, Okamoto T, Lisanti MP (1997). Recombinant expression of caveolin-1 in oncogenically transformed cells abrogates anchorage-independent growth.

[29] Kim YN, Dam P, Bertics PJ (2002). Caveolin-1 phosphorylation in human squamous and epidermoid carcinoma cells: dependence on ErbB1 expression and Src activation. Exp Cell Res.

[30] Orlichenko L, Huang B, Krueger E, McNiven MA (2006). Epithelial growth factor-induced phosphorylation of caveolin 1 at tyrosine 14 stimulates caveolae formation in epithelial cells. J Biol Chem.

[31] Mineo C, Gill GN, Anderson RG (1999). Regulated migration of epidermal growth factor receptor from caveolae. J Biol Chem.

[32] Agelaki S, Spiliotaki M, Markomanolaki H, Kallergi G, Mavroudis D, Georgoulias V, Stournaras C (2009). Caveolin-1 regulates EGFR signaling in MCF-7 breast cancer cells and enhances gefitinib-induced tumor cell inhibition. Cancer Biol Ther.

[33] Klessner JL, Desai BV, Amargo EV, Getsios S, Green KJ (2009). EGFR and ADAMs cooperate to regulate shedding and endocytic trafficking of the desmosomal cadherin desmoglein 2. Mol Biol Cell.

[34] Lorch JH, Klessner J, Park JK, Getsios S, Wu YL, Stack MS, Green KJ (2004). Epidermal growth factor receptor inhibition promotes desmosome assembly and strengthens intercellular adhesion in squamous cell carcinoma cells. J Biol Chem.

[35] Gaudry CA, Palka HL, Dusek RL, Huen AC, Khandekar MJ, Hudson LG, Green KJ (2001). Tyrosine-phosphorylated plakoglobin is associated with desmogleins but not desmoplakin after epidermal growth factor receptor activation. J Biol Chem.

[36] Hackel PO, Zwick E, Prenzel N, Ullrich A (1999). Epidermal growth factor receptors: critical mediators of multiple receptor pathways. Curr Opin Cell Biol.

[37] Chung J, Uchida E, Grammer TC, Blenis J (1997). STAT3 serine phosphorylation by ERK-dependent and -independent pathways negatively modulates its tyrosine phosphorylation. Mol Cell Biol.

[38] Biscardi JS, Maa MC, Tice DA, Cox ME, Leu TH, Parsons SJ (1999). c-Src-mediated phosphorylation of the epidermal growth factor receptor on Tyr845 andTyr1101 is associated with modulation of receptor function. J Biol Chem.

[39] Olayioye MA, Beuvink I, Horsch K, Daly JM, Hynes NE (1999). ErbB receptor-induced activation of stat transcription factors is mediated by Src tyrosine kinases. J Biol Chem.

[40] Keilhack H, Tenev T, Nyakatura E, Godovac-Zimmermann J, Nielsen L, Seedorf K, Böhmer FD (1998). Phosphotyrosine 1173 mediates binding of the protein-tyrosine phosphatase SHP-1 to the epidermal growth factor receptor and attenuation of receptor signaling. J Biol Chem.

[41] Suomela S, Elomaa O, Skoog T, Ala-aho R, Jeskanen L, Pärssinen J, Latonen L, Grénman R, Kere J, Kähäri VM, Saarialho-Kere U (2009). CCHCR1 is up-regulated in skin cancer and associated with EGFR expression. PLoS One.

[42] Graness A, Hanke S, Boehmer FD, Presek P, Liebmann C (2000). Protein-tyrosine-phosphatase-mediated epidermal growth factor (EGF) receptor transinactivation and EGF receptor-independent stimulation of mitogen-activated protein kinase by bradykinin in A431 cells. Biochem J.

[43] Biscardi JS, Maa MC, Tice DA, Cox ME, Leu TH, Parsons SJ (1999). c-Src-mediated phosphorylation of the epidermal growth factor receptor on Tyr845 and Tyr1101 is associated with modulation of receptor function. J Biol Chem.

[44] Quadros MR, Peruzzi F, Kari C, Rodeck U (2004). Complex regulation of signal transducers and activators of transcription 3 activation in normal and malignant keratinocytes. Cancer Res.

[45] Xiong A, Yang Z, Shen Y, Zhou J, Shen Q (2014). Transcription Factor STAT3 as a Novel Molecular Target for Cancer Prevention. Cancers (Basel).

[46] Barnes CJ, Bagheri-Yarmand R, Mandal M, Yang Z, Clayman GL, Hong WK, Kumar R (2003). Suppression of epidermal growth factor receptor, mitogen-activated protein kinase, and Pak1 pathways and invasiveness of human cutaneous squamous cancer cells by the tyrosine kinase inhibitor ZD1839 (Iressa). Mol Cancer Ther.

[47] Kansra S, Stoll SW, Johnson JL, Elder JT (2005). Src family kinase inhibitors block amphiregulin-mediated autocrine ErbB signaling in normal human keratinocytes. Mol Pharmacol.

[48] Byers LA, Sen B, Saigal B, Diao L, Wang J, Nanjundan M, Cascone T, Mills GB, Heymach JV, Johnso nFM (2009). Reciprocal regulation of c-Src and STAT3 in non-small cell lung cancer. Clin Cancer Res.

[49] Lajoie P, Nabi IR (2007). Regulation of raft-dependent endocytosis. J Cell Mol Med.

[50] Li S, Couet J, Lisanti MP (1996). Src tyrosine kinases, Galpha subunits, and H-Ras share a common membrane-anchored scaffolding protein, caveolin. Caveolin binding negatively regulates the auto-activation of Src tyrosine kinases. J Biol Chem.

[51] Hell SW, Wichmann J (1994). Breaking the diffraction resolution limit by stimulated emission: stimulated-emission-depletion fluorescence microscopy. Opt Lett.

[52] Craven RJ, Lightfoot H, Cance WG (2003). A decade of tyrosine kinases: from gene discovery to therapeutics. Surg Oncol.

[53] Jost M, Huggett TM, Kari C, Rodeck U (2001). Matrix-independent survival of human keratinocytes through an EGF receptor/MAPK-kinase-dependent pathway. Mol Biol Cell.

[54] Andl CD, Mizushima T, Nakagawa H, Oyama K, Harada H, Chruma K, Herlyn M, Rustgi AK (2003). Epidermal growth factor receptor mediates increased cell proliferation, migration, and aggregation in esophageal keratinocytes *in vitro* and *in vivo*. J Biol Chem.

[55] Chitaev NA, Troyanovsky SM (1997). Direct Ca2+ dependent heterophilic interaction between desmosomal cadherins, desmoglein and desmocollin, contributes to cell adhesion. J Cell Biol.

[56] Jiang K, Rankin CR, Nava P, Sumagin R, Kamekura R, Stowell SR, Feng M, Parkos CA, Nusrat A (2014). Galectin-3 regulates desmoglein-2 and intestinal epithelial intercellular adhesion. J Biol Chem.

[57] Irwin ME, Bohin N, Boerner JL (2011). Src family kinases mediate epidermal growth factor receptor signaling from lipid rafts in breast cancer cells. Cancer Biol Ther.

[58] Goh LK, Huang F, Kim W, Gygi S, Sorkin A (2010). Multiple mechanisms collectively regulate clathrin-mediated endocytosis of the epidermal growth factor receptor. J Cell Biol.

[59] Roepstorff K, Grøvdal L, Grandal M, Lerdrup M, van Deurs B (2008). Endocytic downregulation of ErbB receptors: mechanisms and relevance in cancer. Histochem Cell Biol.

[60] Madshus IH, Stang E (2009). Internalization and intracellular sorting of the EGF receptor: a model for understanding the mechanisms of receptor trafficking. J Cell Sci.

[61] Lambert S, Vind-Kezunovic D, Karvinen S, Gniadecki R (2006). Ligand-independent activation of the EGFR by lipid raft disruption. J Invest Dermatol.

[62] Jans R, Atanasova G, Jadot M, Poumay Y (2004). Cholesterol depletion upregulates involucrin expression in epidermal keratinocytes through activation of p38. J Invest Dermatol.

[63] Mathay C, Pierre M, Pittelkow MR, Depiereux E, Nikkels AF, Colige A, Poumay Y (2011). Transcriptional profiling after lipid raft disruption in keratinocytes identifies critical mediators of atopic dermatitis pathways. J Invest Dermatol.

[64] Goetz JG, Lajoie P, Wiseman SM, Nabi IR (2008). Caveolin-1 in tumor progression: the good, the bad and the ugly. Cancer Metastasis Rev.

[65] Masuda H, Zhang D, Bartholomeusz C, Doihara H, Hortobagyi GN, Ueno NT (2012). Role of epidermal growth factor receptor in breast cancer. Breast Cancer Res Treat.

[66] Antonarakis ES, Carducci MA, Eisenberger MA (2010). Novel targeted therapeutics for metastatic castration-resistant prostate cancer. Cancer Lett.

[67] Haghgoo SM, Allameh A, Mortaz E, Garssen J, Folkerts G, Barnes PJ, Adcock IM (2015). Pharmacogenomics and targeted therapy of Cancer: Focusing on Non-small cell lung Cancer. Eur J Pharmacol.

[68] Zhang S, Yu D (2012). Targeting Src family kinases in anti-cancer therapies: turning promise into triumph. Trends Pharmacol Sci.

[69] Liu J, Wang H, Qu A, Li J, Zhao Y, Wang J (2013). Combined effects of C225 and 125-iodine seed radiation on colorectal cancer cells. Radiat Oncol.

[70] Reigneau M, Robert C, Routier E, Mamelle G, Moya-Plana A, Tomasic G, Mateus C (2015). Efficacy of neoadjuvant cetuximab alone or with platinum salt and fluorouracil for the treatment of unresectable locally advanced cutaneous squamous cell carcinomas. Br J Dermatol.

[71] Lewis CM, Glisson BS, Feng L, Wan F, Tang X, Wistuba II, El-Naggar AK, Rosenthal DI, Chambers MS, Lustig RA, Weber RS (2012). A phase II study of gefitinib for aggressive cutaneous squamous cell carcinoma of the head and neck. Clin Cancer Res.

[72] Wahl JK, Sacco PA, MaGranahan-Sadler TM, Sauppe LM, Wheelock MJ, Johnson KR (1996). Plakoglobin domains that define its association with the desmosomal cadherins and the classical cadherins: identification of unique and shared domains. J Cell Sci.

[73] Keim SA, Johnson KR, Wheelock MJ, Wahl JKr (2008). Generation and characterization of monoclonal antibodies against the proregion of human desmoglein-2. Hybridoma (Larchmt).

[74] Boukamp P, Petrussevska RT, Breitkreutz D, Hornung J, Markham A, Fusenig NE (1988). Normal keratinization in a spontaneously immortalized aneuploid human keratinocyte cell line. J Cell Biol.

[75] Schoop VM, Mirancea N, Fusenig NE (1999). Epidermal organization and differentiation of HaCaT keratinocytes in organotypic coculture with human dermal fibroblasts. J Invest Dermatol.

